# Preliminary validation of the self-report measure assessing experiences of negative independent and dependent event frequency in Japanese university students

**DOI:** 10.1007/s10942-022-00469-9

**Published:** 2022-07-30

**Authors:** Akira Hasegawa, Shin-ichi Oura, Tetsuya Yamamoto, Yoshihiko Kunisato, Yoshikazu Fukui

**Affiliations:** 1 Department of Psychology, International University of Health and Welfare, 4-1-26 Akasaka, Minato-ku, Tokyo, 107-8402 Japan; 2 Faculty of Human Relations, Tokai Gakuin University, 5-68 Naka-kirino, Kakamigahara City, Gifu 504-8511 Japan; 3 Graduate School of Technology, Industrial and Social Sciences, Tokushima University, 1-1, Minamijosanjima-cho, Tokushima, 770-8502 Japan; 4 Department of Psychology, School of Human Sciences, Senshu University, 2-1-1, Higashimita, Tama-ku, Kawasaki-shi, Kanagawa 214-8580 Japan; 5 Faculty of Letters, Konan University, 8-9-1 Okamoto, Higashinada-ku, Kobe, Hyougo 658-8501 Japan

**Keywords:** Stress generation, Negative events, Depression, Reassurance-seeking, Inattention

## Abstract

**Background:**

In the past, different stress generation studies have used self-report measures comprising different items to assess each category of negative events. Moreover, the validity of these scales has not been adequately investigated. Therefore, we developed a self-report measure dedicated to assessing experiences of negative interpersonal dependent events, negative non-interpersonal dependent events, and negative independent events in university students, which was named the Negative Independent/Dependent Events Scale.

**Methods:**

Japanese undergraduate students (N = 247; mean age = 19.18 years, *SD* = 3.08) responded to the Negative Independent/Dependent Events Scale, which had items selected for adequate content validity. They also responded to self-report measures of depressive symptoms, reassurance-seeking behaviors, inattention, and lack of perseverance.

**Results:**

All the negative events subscales had moderate positive correlations with depressive symptoms. In addition, the negative interpersonal dependent events subscale showed a moderate positive correlation with reassurance-seeking behaviors, and the negative non-interpersonal dependent events subscale showed a strong positive correlation with inattention. Furthermore, the negative non-interpersonal dependent events subscale was more strongly correlated with inattention than the other two negative events subscales. In contrast, the negative interpersonal dependent events subscale was more strongly correlated with reassurance-seeking behaviors than with the negative independent events subscale but not more strongly than with the negative non-interpersonal dependent events subscale.

**Conclusions:**

These findings indicated the acceptable construct validity of the Negative Independent/Dependent Events Scale. However, further research is necessary to establish the discriminant validity of the negative interpersonal dependent events subscale and the negative non-interpersonal dependent events subscale.

**Supplementary Information:**

The online version contains supplementary material available at 10.1007/s10942-022-00469-9.

## Stress generation hypothesis

Since the seminal work by Hammen (1991), researchers have assumed that depression-prone individuals are not simply passive recipients of negative life events but active agents in the creation of such events.^1^ Longitudinal studies based on this assumption named the *stress generation hypothesis* (Hammen, 1991) have shown that individuals with depressive disorders or subclinical depressive symptoms experience negative events more frequently than their non-depressed counterparts (Liu & Alloy, 2010, for review).

Furthermore, studies suggest that individuals with depressogenic characteristics such as negative cognitive styles, ruminative response styles, neuroticism, maladaptive attachment styles, and excessive reassurance-seeking, experience frequent negative events (Liu & Alloy, 2010, for review). The mechanism by which these traits lead to stress generation remains unclear. However, behavioral styles might mediate relationships between intrinsic variables such as cognitive factors and stress generation because only expressed behaviors can change the environment (Liu 2013). For example, it is known that excessive reassurance-seeking mediates the association between rumination and stress generation (Stroud et al., 2018). Recent research has also indicated biological bases for stress generation, which include the serotonin transporter-linked polymorphic region genotype, a critical molecule that regulates serotonergic neurotransmission at the synaptic cleft and influences emotions and stress responses (Harkness et al., 2015; Starr et al., 2012), and stress-reactive respiratory sinus arrhythmia (Hamilton & Alloy, 2017).

Negative life events generated by the self might predict future increases in depressive symptoms (e.g., Belmans et al., 2019; Flynn et al., 2010; Flynn & Rudolph, 2011; Hankin et al., 2010; Snyder & Hankin, 2016), and the recurrence of major depression (Bos et al., 2007). Therefore, the stress generation hypothesis can explain person-environment interactions exacerbating depression.

## Assessment in stress generation research context

Stress generation studies have often classified negative events into *independent events* occurring outside the individual’s control, such as the death of a relative or friend, and *dependent events* occurring under the influence of the individual’s control, such as interpersonal conflicts. Negative events have also been classified as interpersonal and non-interpersonal events, with the latter including work-related and academic stressors (Hammen & Shih, 2008; Liu & Alloy, 2010). It has been reported that individuals vulnerable to depression are likely to experience a higher rate of dependent events, particularly within the interpersonal domain rather than independent events (Liu & Alloy, 2010, for review). Therefore, it is crucial for stress generation studies to use assessment tools to assess negative independent and negative dependent events separately and identify these events as related to interpersonal or non-interpersonal domains.

Specific studies on stress generation have assessed the experiences of negative events using self-report measures (e.g., Bouchard & Shih 2013; Hankin et al., 2010; Liu & Kleiman, 2012), and other studies have assessed them using interviews (e.g., Flynn & Rudolph 2011; Hammen, 1991; Harkness et al., 2015). Assessments using self-report measures have several shortcomings, including the contamination of subjective appraisal of negative events in assessing the actual occurrence of such events and difficulties in collecting detailed contextual information on events for judging whether they are dependent on the individual’s control or not, and whether they are interpersonal or not. However, self-report measures of negative events have specific strengths, including the short response time compared to interviews that might take over one hour (Liu, 2013). Therefore, self-report measures are appropriate when researchers use multiple measures for assessing many constructs that take an extended time, and negative events are only one of the constructs of interest. In addition, self-report measures can be used in online surveys, which is not the case with interviews.

## Limitations of current self-report measures of negative events

Self-report measures used in previous stress generation studies have specific limitations. Firstly, investigators independently categorized negative events in the scales, which were not explicitly designed to test the stress generation hypothesis, as independent or dependent events and interpersonal or non-interpersonal events. Therefore, different items of the same scale have been used to assess each category of negative events in different studies. For example, Hankin et al. (2010) classified 57 items in the Adolescent Life Events Questionnaire (ALEQ; Hankin & Abramson 2002) into 26 interpersonal dependent events, 11 non-interpersonal dependent events, and 13 independent events. Moreover, they excluded 7 items because these items were neither interpersonal nor non-interpersonal. Auerbach et al. (2010), who used the ALEQ composed of the identical 57 items, classified 29 items as interpersonal dependent events. On the other hand, Belmans et al. (2019) used only 18 items as interpersonal dependent events, although it is uncertain whether they used the identical ALEQ items as Hankin et al. (2010) and Auerbach et al. (2010). Using different items to assess each category of negative event experiences has made it difficult to compare findings between studies.

Furthermore, the validity of currently available negative events scales has not been adequately investigated in the stress generation research context. Researchers have merely selected negative interpersonal dependent event items, negative non-interpersonal dependent event items, and negative independent event items by considering their content validity. However, the construct validity of these scales has not been examined to date. Therefore, a need remains to develop a scale with high construct validity that can specifically assess each category of negative events.

To our knowledge, there has been no attempt in Japan to assess negative dependent events and negative independent events separately. For example, Hasegawa et al. (2022a) examined whether response inhibition deficits increased rumination through the generation of negative interpersonal events in Japanese university students. However, they did not use negative interpersonal dependent event items selected in the stressors scale in their analyses because the interpersonal events subscale they used consisted of only 15 items. Developing a measure for specifically assessing different event categories in the context of stress generation would help identify determinants and consequences of stress generation.

University systems vary from country to country. In addition, Markus & Kitayama (1991) suggested that Western and Asian people have strikingly different self-construals. They theorized that Western people see the self as separate from others, and their self-esteem is dependent on the ability to express the self and validate internal attributes. On the other hand, Asian people see the self as interdependent with others in significant social units, and their self-esteem is dependent on the ability to maintain harmony within the social context (see also Kitayama et al., 2009; Park et al., 2016). Because of these differences, we considered it desirable to develop a scale assessing negative events that Japanese university students frequently experience rather than merely translating scales developed in other countries. Furthermore, it is necessary to develop a scale of negative events suitable for peoples’ life stage because negative events that people tend to experience differ depending on their life stage.

## Purposes and hypotheses

Considering the above issues, we developed a scale to separately assess the experiences of negative interpersonal dependent events, negative non-interpersonal events, and negative independent events in Japanese university students. A scale for assessing negative events experienced by university students was developed because university students are highly targeted populations in stress generation research (e.g., Bouchard & Shih 2013; Flynn et al., 2010; Joiner et al., 2005; Liu & Kleiman, 2012). This scale was named the Negative Independent/Dependent Events Scale. Items with high content validity for the three subscales were selected among an item pool of existing scales. We also tested the scale’s construct validity.

We tested the following hypotheses to examine the construct validity of the Negative Independent/Dependent Events Scale. Negative interpersonal dependent events, negative non-interpersonal events, and negative independent events are stressful. Therefore, we hypothesized a moderate or a strong positive correlation between all the event categories and depressive symptoms (Hypothesis 1).

In addition, a previous study has indicated that reassurance-seeking behaviors were specifically associated with negative interpersonal dependent events (Stroud et al., 2018). Therefore, we hypothesized a moderate or strong positive correlation between negative interpersonal dependent events and reassurance-seeking behaviors, which is stronger than the correlation between reassurance-seeking behaviors and negative non-interpersonal dependent events or negative independent events (Hypothesis 2).

Furthermore, university students with increased inattention symptoms caused by ADHD are likely to have academic problems (Norwalk et al., 2009; Pope, 2010; Schwanz et al., 2007). Academic problems are a large part of negative non-interpersonal dependent events experienced by university students (see items of negative non-interpersonal dependent events in the Results section). Therefore, we hypothesized a moderate or strong positive correlation between negative non-interpersonal dependent events and inattention and that the correlation between negative non-interpersonal dependent events and inattention is stronger than the correlations between other types of negative events and inattention (Hypothesis 3).

Similarly, university students with high lack of perseverance scores, which is a subdimension of self-reported impulsivity reflecting not following through with a task (Lynam et al., 2006), might have difficulties in completing academic assignments. Therefore, it was hypothesized that a moderate or a strong positive correlation is observed between negative non-interpersonal dependent events and the lack of perseverance, and that the correlation between negative non-interpersonal dependent events and the lack of perseverance is stronger than those between other negative events and the lack of perseverance (Hypothesis 4).

Finally, we attempted to reduce the items of the Negative Independent/Dependent Events Scale because a short scale would allow assessing participants’ experiences of negative events in a short time. We selected items with a relatively normal distribution of scores and high construct validity for the short scale. This study was conducted under the unusual situation of the global COVID-19 pandemic. Therefore, we could only present preliminary items for the short scale.

## Method

### Participants

Japanese undergraduate students at Tokai Gakuin University and Tokushima Universities in Japan (*N* = 270) participated in this study. Participants were recruited in their psychology classes. We collected as much data as possible from July to August 2020. All participants who agreed to participate in the paper and pencil survey completed a packet of self-report measures in the classroom after psychology classes.

Data of participants with missing values on any questionnaire, or inappropriate responses, including identical responses to all the items in each questionnaire, were excluded from the analysis. The final sample comprised 247 students (153 men, 93 women, and 1 unreported gender; mean age = 19.18 years, *SD* = 3.08, age-range 18–48 years).

### Measures

***Negative Independent/Dependent Events Scale***. This self-report measure was originally developed for this study. We took the following steps to develop a brief scale consisting of items with high content validity.

An associate professor in Japan, specializing in psychology (AH) developed an initial pool of 286 items of negative events by reviewing the literature (Hashimoto, 1997, 2005; Hisata & Niwa, 1987; Kanner et al., 1981; Kikushima, 1999, 2002; Kohn et al., 1990; Miura & Kawaoka, 2008; Nishino et al., 2009; Okayasu et al., 1992; Sakamoto & Kambara, 1998; Shimono & Hasegawa, 2018; Takahashi, 2013; Takahira, 1998; Toyama & Sakurai, 1999). AH and a full-time lecturer specializing in psychology (SO) selected the items suitable for assessing negative interpersonal dependent events, negative non-interpersonal dependent events, and negative independent events from these 286 items. As a result, we selected 80 items; 29 negative interpersonal dependent event items, 22 negative non-interpersonal dependent event items, and 29 negative independent events.

Then, three different associate professors, a postdoctoral fellow, two graduate students studying for a Master’s degree, all of them specializing in psychology, and two clinical psychologists with Master’s degrees, were asked to rate the 80 items described above. Each event was rated on the following dimensions: (1) the relationship to interpersonal events rated on a 5–point scale ranging from 1 (*not an interpersonal event at all*) to 5 (*highly interpersonal event*), and (2) the relationship to students’ behaviors, personality, and attitudes (i.e., a dependence rating) rated on a 5-point scale ranging from 1 (*not related at all* or *completely independent)* to 5 (*highly related*). We calculated the mean scores of the two ratings for each item. As a result, 25 items among the negative interpersonal dependent event items with a mean score of 3.5 or above for the interpersonal events rating and a mean score of 3 or more for the dependence rating were selected as the final negative interpersonal dependent event items. Similarly, 14 items among the negative non-interpersonal dependent event items with a mean score of 2.5 or less for interpersonal events rating and 3 or more for dependence ratings were selected as the final items. Furthermore, 20 items among negative independent events items with a mean score of 2 or less for dependence rating were selected as the final items.

Participants were requested to respond to the extent to which they had experienced each event in the last eight weeks using a rating scale ranging from 1 (*never*) to 4 (*often*). Participants were instructed to report the experiences of each negative event in the past 8 weeks, consistent with the interval of another 8-weeks longitudinal study that we have planned.^2^

***Beck Depression Inventory-Second Edition *(**Beck et al., 1996). This scale assesses the severity of depressive symptoms experienced in the past two weeks. Participants respond to 21 items using a 0–3 scale, with higher scores indicating more severe depressive symptoms. We used the Japanese translation of the BDI-II by Kojima & Furukawa (2003). The BDI-II showed good reliability and validity as the original version (Beck et al., 1996) and the Japanese version (Kojima & Furukawa, 2003). This study showed the excellent internal consistency of the scale (*α* = 0.93).

***Revised Japanese Version of the Reassurance-Seeking Scale *(**Katsuya 2004**).** This scale is designed to assess behaviors and the desire to seek reassurance. This scale, developed with a Japanese university student sample, indicated good reliability and construct validity (Katsuya, 2004). The reassurance-seeking behaviors subscale, which is composed of 6 items, was used in this study. Each item was rated on a 7-point rating scale anchored between 1 (*not at all true of me*) and 7 (*entirely true of me*). The sufficient internal consistency of the scale was demonstrated in this study (*α* = 0.82).

***Adult ADHD Self-Report Scale *(**Kessler et al., 2005). This is a self-report measure designed to assess adults’ ADHD symptoms. The Japanese translation of the scale by Takeda et al. (2017) was used in this study. The Adult ADHD Self-Report Scale has demonstrated good reliability and construct validity as the original version (Adler et al., 2006; Kessler et al., 2007) and the Japanese version (Takeda et al., 2017). The scale is composed of two subscales, and we only used the inattention subscale. The scale inquires the frequency of symptoms over the past six months using 9 items, which are rated on a 5-point rating scale anchored between 0 (*never*) and 4 (*very often*). The satisfactory internal consistency of the inattention subscale was demonstrated in this study (*α* = 0.84).

***UPPS-P Impulsive Behavior Scale *(**Lynam et al., 2006). This is a measure assessing five impulsivity-related traits. We used the Japanese translation by Hasegawa et al. (2018). The UPPS-P Impulsive Behavior Scale has reported good reliability and construct validity as the original version (Cyders, 2013; Cyders et al., 2007; Whiteside et al., 2005), and the Japanese version (Hasegawa et al., 2018). Only the lack of perseverance subscale was used in this study. The 10 items of this subscale were rated on a 4-point rating scale anchored between 1 (*disagree strongly*) and 4 (*agree strongly*). This study demonstrated the acceptable internal consistency of this subscale (*α* = 0.76).

### Procedure

This study was conducted from July to August 2020. Since February 2020, COVID-19 had spread in Japan, and all classes in Tokai Gakuin University and Tokushima University had been conducted online until May 2020. When this study was conducted, students in both universities took classes on campus for at least six weeks.

We explained the study verbally and in writing to the students before participation. We also informed them that they could withdraw from the study at any time, including before and during responding to the questionnaire. Only students that agreed to participate in the study responded to the questionnaires, at their own pace. The participants did not receive any compensation for participating in the study. The Ethics Committee of Tokai Gakuin University approved this study.

### Statistical analysis

Analyses were conducted on raw data and allowed for missing data. Descriptive statistics were conducted using SPSS ver. 23 (IBM Corporation). The *Z*-test and post hoc power of correlations was computed using R ver. 4.0.0. Other analyses were conducted using Mplus 8.3 (Muthén & Muthén, 1998–2017). Zero-order Pearson’s correlations were computed between each measure, and the *Z*-test to compare two dependent correlations was conducted using the procedure described by Hittner et al. (2003). Multivariate outliers were checked using Cook’s D values of each correlation among all study variables.^3^ None of the participants showed a correlation having a Cook’s D value greater than one.

Missing data were handled with multiple imputations using Bayesian analysis when conducting correlation analyses. We inputted all the items of the scales described in the Measures section, and 20 data sets were generated and used for the analyses. We refer to the magnitude of the correlations, using Cohen’s (1988) classification (*r* < .10: negligible association; 0.10 ≤ *r* < .30: weak association; 0.30 ≤ *r* < .50: moderate association; *r* ≥ .50: strong association). When comparing two dependent correlations, we applied the Bonferroni correction to divide the significance level of the *p*-value by three (the number of all pairs of correlations; *p* = .05/3 = 0.017). We computed the post hoc power of correlations. Results showed that we could detect correlations of 0.18 with 80% power set at *α* = 0.05 in a two-tailed test.^4^

Performing factor analysis on scale items is useful when developing a scale composed of multiple subscales. However, because the Negative Independent/Dependent Events Scale assesses the frequency of different negative life events, rather than multiple indicators of a particular construct, it is incorrect to assume that the score of each item is influenced by a latent construct (see Snyder et al., 2019). Therefore, we did not conduct factor analysis on this scale’s items to extract the latent construct from each item score. We calculated the subscale scores for negative interpersonal dependent events, negative non-interpersonal dependent events, and negative independent events by simply summing the score of each item that we selected a priori for each subscale.

West et al. (1995) suggested that an absolute skewness value of two or over and/or an absolute kurtosis value of four or over indicate skewed distributions. We considered the distorted distribution of scale scores or items according to this suggestion.

## Results

### Descriptive statistics

Descriptive statistics of each measure are shown in Table 1. All the study variables’ scores, including the three subscales of negative events, were normally distributed. Alpha coefficients were 0.91 for negative interpersonal dependent events, 0.80 for negative non-interpersonal dependent events, and 0.81 for negative independent events. Table S1 shows male and female students’ descriptive statistics of the measures. Student *t*-tests indicated that female university students scored higher on negative non-interpersonal dependent events, depressive symptoms, reassurance-seeking behaviors, and inattention than male students (see Table S1).

### Construct validity of the negative Independent/Dependent events Scale

Correlations between each measure are shown in Table 2. Results indicated strong, significant positive correlations among negative interpersonal dependent events, negative non-interpersonal dependent events, and negative independent events. All the negative events subscales indicated significant and moderate positive correlations with depressive symptoms and reassurance-seeking behaviors. Negative non-interpersonal dependent events showed a strong positive correlation with inattention, whereas negative interpersonal dependent events and negative independents events displayed moderate correlations with inattention. Furthermore, negative interpersonal dependent events and negative non-interpersonal dependent events indicated a significant but weak positive correlation with the lack of perseverance, whereas negative independent events showed a non-significant correlation with the lack of perseverance.

**Table 1:** Descriptive statistics of study measures

	*n*		*M*		*SD*		Range		Skewness					Kurtosis			
									Statistic		*SE*			Statistic		*SE*	
Negative interpersonal dependent events	240		38.97		11.18		25–70		0.62		0.16			–0.61		0.31	
Negative non-interpersonal dependent events	243		25.22		6.05		14–48		0.67		0.16			0.46		0.31	
Negative independent events	243		32.58		7.63		20–60		0.77		0.16			0.26		0.31	
Depressive symptoms	240		13.65		10.63		0–52		1.12		0.16			1.13		0.31	
Reassurance-seeking behaviors	244		22.58		8.08		6–40		–0.26		0.16			–0.48		0.31	
Inattention	242		15.68		5.77		0–34		0.30		0.16			0.54		0.31	
Lack of perseverance	246		23.71		4.90		12–39		0.10		0.16			0.11		0.31	

Note: Negative interpersonal dependent events, negative non–interpersonal dependent events, and negative independent events are subscale scores on the Negative Independent/Dependent Events Scale. Depressive symptoms mean total score on the Beck Depression Inventory-Second Edition. Reassurance-seeking behaviors means a subscale score on the Revised Japanese Version of the Reassurance-Seeking Scale. Inattention means a subscale score on the Adult ADHD Self-Report Scale. Lack of perseverance means a subscale score on UPPS-P Impulsive Behavior Scale

The *Z*-test showed that the correlations of both negative dependent events with depressive symptoms were stronger than that of negative independent events with depressive symptoms (*qs* > 0.14, *zs* > 2.73, *ps* < 0.007). However, difference in correlations with depressive symptoms was not significant between negative interpersonal dependent events and negative non-interpersonal events (*q* = 0.02, *z* = 0.34, *p* = .732). The correlation of negative interpersonal dependent events with reassurance-seeking behaviors was stronger than that of negative independent events (*q* = 0.15, *z* = 2.67, *p* = .008), but the differences in the correlations with reassurance-seeking behaviors was non-significant between other pairs of negative events (*qs* < 0.11, *zs* < 1.87, *ps* > 0.061). The correlation of negative non-interpersonal dependent events with inattention was stronger than those of the other two negative events (*qs* > 0.22, *zs* > 3.84, *ps* < 0.001), whereas the difference in the correlations with inattention was not significant between negative interpersonal dependent events and negative independent events (*q* = 0.09, *z* = 1.59, *p* = .112). The differences in the correlation with lack of perseverance were not significant between any pair of negative events (*q* < 0.13, *z* < 2.33, *p* > .020). Table S2 shows the results of the *Z* tests in detail.

### Selection of appropriate items for the short scale

We conducted supplementary analyses to identify the appropriate items for each subscale of a short scale. Each item was evaluated on their score distribution because items with skewed distributions are inappropriate for assessing individual differences in experiencing different event categories. We considered items with skewness of 2.00 or over or kurtosis of 4.00 or over as inappropriate. In addition, we evaluated each item for correlations with reassurance-seeking behaviors and inattention. As described above, the hypothesized correlation between lack of perseverance and non-interpersonal dependent events was weak (see Table 2). Inspecting the lack of perseverance subscale suggested that the subscale reflects constructs other than the lack of perseverance (see the Discussion for details). Therefore, we did not use the lack of perseverance subscale to evaluate the appropriateness of the shortened Negative Independent/Dependent Events Scale items. We considered that items having a correlation of less than 0.20 with reassurance-seeking behaviors were inappropriate for assessing negative interpersonal dependent events. Moreover, items with a correlation of less than 0.20 with inattention were considered inappropriate for assessing negative non-interpersonal dependent events. Furthermore, items having correlations of 0.20 or over with reassurance-seeking behaviors and inattention were considered inappropriate for assessing negative independent events.

Among the items, 15 items were highly skewed (6 negative interpersonal dependent events items, 2 negative non-interpersonal dependent events items, and 7 negative independent events items; see Tables 3, 4 and 5). Among the remaining items, 4 negative interpersonal dependent events items had correlations of less than 0.20 with reassurance-seeking behaviors, and 4 negative independent events items had correlations of 0.20 or more with reassurance-seeking behaviors and inattention (see Tables 3, 4 and 5).

**Table 2:** Correlations between variables (N = 247)

	1		2		3		4		5		6	
1. Negative interpersonal dependent events	–											
2. Negative non-interpersonal dependent events	0.69		–									
	[0.63, 0.76]											
3. Negative independent events	0.69		0.68		–							
	[0.62, 0.75]		[0.61, 0.74]									
4. Depressive symptoms	0.45		0.47		0.33		–					
	[0.35, 0.55]		[0.37, 0.57]		[0.22, 0.44]							
5. Reassurance-seeking behaviors	0.45		0.42		0.33		0.43		–			
	[0.35, 0.55]		[0.32, 0.52]		[0.22, 0.44]		[0.33, 0.54]					
6. Inattention	0.45		0.61		0.38		0.56		0.40		–	
	[0.35, 0.55]		[0.53, 0.69]		[0.26, 0.49]		[0.47, 0.65]		[0.29, 0.51]			
7. Lack of perseverance	0.16		0.22		0.10		0.19		0.10		0.38	
	[0.04, 0.28]		[0.10, 0.34]		[–0.02, 0.23]		[0.07, 0.31]		[–0.03, 0.22]		[0.27, 0.49]	

Note: Absolute correlations greater than 0.16 are significant at *p* < .05. Numbers in parentheses indicate 95% confidence intervals

**Table 3:** Negative interpersonal dependent events items (English translations)

	Reassurance-seeking behaviors			Inattention		
I had a quarrel with my family member.	0.29		[0.18, 0.41]	0.33		[0.22, 0.44]
I disagreed with a close person such as my friend or a lover.	0.36		[0.25, 0.47]	0.26		[0.14, 0.38]
I was criticized or teased by my friends and colleagues about what I did.	0.26		[0.14, 0.38]	0.11		[–0.01, 0.24]
I was criticized by others where I wasn’t.	0.25		[0.13, 0.37]	0.19		[0.06, 0.31]
Others were worried due to my wrong behaviors.	0.33		[0.22, 0.44]	0.33		[0.21, 0.44]
Others said or acted as if they did not trust me.	0.37		[0.26, 0.48]	0.29		[0.18, 0.41]
I hurt others.	0.33		[0.21, 0.44]	0.36		[0.25, 0.47]
I was pointed out or warned by my superiors that I had a bad attitude.	0.12		[–0.00, 0.24]	0.21		[0.09, 0.33]
I offended others.	0.37		[0.27, 0.48]	0.36		[0.25, 0.47]
I was left out of my friends.	0.17		[0.05, 0.29]	0.28		[0.16, 0.40]
I was at the mercy of an acquaintance.	0.31		[0.19, 0.42]	0.33		[0.21, 0.44]
I got into the troubles and problems of my friends.	0.19		[0.07, 0.31]	0.08		[–0.05, 0.21]
Although I tried to get the attention of a person of the opposite gender, I was treated cold by him/her.	0.23		[0.11, 0.35]	0.10		[–0.02, 0.23]
I had a quarrel with or had a terrible relationship with my superior.	0.12		[–0.00, 0.25]	0.11		[–0.02, 0.24]
I couldn’t convey what I wanted to say to others.	0.40		[0.29, 0.51]	0.42		[0.32, 0.52]
I was ridiculed by a friend	0.24		[0.13, 0.36]	0.26		[0.15, 0.38]
My relationship got worse at my part-time job	0.24		[0.12, 0.36]	0.15		[0.03, 0.28]
I couldn’t get along with my friends.	0.25		[0.14, 0.37]	0.30		[0.18, 0.42]
When I pointed out other about his/her bad behaviors, I was counterattacked.	0.17		[0.05, 0.29]	0.18		[0.06, 0.30]
I had a fight or a quarrel with a close person such as friends and a lover.	0.26		[0.15, 0.38]	0.20		[0.08, 0.32]
I broke up with my lover, or was turned down by a favorite person.	0.06		[–0.06, 0.19]	0.07		[–0.05, 0.20]
I was ignored by or turned away from my friends.	0.23		[0.11, 0.35]	0.25		[0.13, 0.38]
I bothered others.	0.36		[0.25, 0.47]	0.44		[0.34, 0.54]
I had a terrible relationship with my friend.	0.20		[0.08, 0.32]	0.24		[0.13, 0.36]
I disagreed with my family.	0.25		[0.13, 0.36]	0.37		[0.26, 0.48]

Note: Items with underlines means those with skewness of 2.00 or more, or kurtosis of 4.00 or more. Items with a gray marker mean that their correlations with reassurance-seeking behaviors were less than 0.20. Absolute correlations greater than 0.15 are significant at *p* < .05. Numbers in parentheses indicate 95% confidence intervals

**Table 4:** Negative non-interpersonal dependent events items (English translations)

	Reassurance-seeking behaviors			Inattention		
It took a long time to finish the assignments such as reports.	0.15		[0.02, 0.27]	0.33		[0.21, 0.44]
I could not finish assignments such as reports by the due date.	0.09		[–0.04, 0.21]	0.27		[0.15, 0.39]
I wasn’t satisfied with my presentation.	0.33		[0.22, 0.45]	0.36		[0.25, 0.47]
I was arrested for breaking the law including traffic violations and unpaid rides.	0.09		[–0.04, 0.23]	0.01		[–0.14, 0.16]
I left my stuff outside the house.	0.27		[0.15, 0.38]	0.33		[0.21, 0.44]
I broke or lost something important.	0.23		[0.12, 0.35]	0.31		[0.19, 0.42]
I didn’t have enough money, or was in debt.	0.26		[0.15, 0.38]	0.31		[0.20, 0.43]
I could not improve my hobbies or lessons as I expected.	0.21		[0.09, 0.33]	0.32		[0.20, 0.43]
I had bad results in the exam.	0.18		[0.06, 0.30]	0.24		[0.12, 0.35]
I have accumulated tasks that I have to do such as reports.	0.24		[0.12, 0.36]	0.47		[0.37, 0.57]
I was in trouble about getting a job.	0.20		[0.08, 0.32]	0.24		[0.12, 0.36]
The professor gave me a low rating when I made a presentation in class.	0.25		[0.14, 0.37]	0.32		[0.21, 0.43]
My report was badly rated.	0.22		[0.10, 0.34]	0.30		[0.19, 0.42]
I couldn’t go well with my learning, study, graduation, etc.	0.32		[0.20, 0.43]	0.49		[0.40, 0.59]

Note: Items with underlines means those with skewness of 2.00 or more, or kurtosis of 4.00 or more. Items with a gray marker mean that their correlations with inattention were less than 0.20. Absolute correlations greater than 0.15 are significant at *p* < .05. Numbers in parentheses indicate 95% confidence intervals

We summed the scores of the items of each subscale after excluding 23 items described above. Mean scores (*SDs*) of each subscale were 25.81 (8.19) for negative interpersonal dependent events, 22.72 (5.61) for negative non-interpersonal dependent events, and 15.21 (4.17) for negative independent events. Skewness and kurtosis were 0.53 and − 0.56, respectively for negative interpersonal dependent events, 0.64 and 0.49, respectively for negative non-interpersonal dependent events, and 0.54 and − 0.15, respectively for negative independent events. Cronbach’s alphas were 0.90, 0.79, and 0.65, respectively for negative interpersonal dependent events, negative non-interpersonal dependent events, and negative independent events.

We also calculated the correlation coefficients among the three subscales and correlation coefficients of each subscale with reassurance-seeking behaviors, inattention, and lack of perseverance. As shown in Table 6, the correlation among each short scale’s subscale and the correlations of negative independent events subscale with the validity scales were slightly weaker than the original Negative Independent/Dependent Events Scale. The *Z*-test indicated that correlations of both negative dependent events and depressive symptoms were stronger than that of negative independent events (*qs* > 0.20, *zs* > 3.13, *ps* < 0.002), while differences in the correlations of negative interpersonal dependent events and negative non-interpersonal events with depressive symptoms was not significant (*q* = 0.08, *z* = 1.44, *p* = .149). The correlations of both negative dependent events with reassurance-seeking behaviors were stronger than that of negative independent events (*qs* > 0.17, *zs* > 2.58, *ps* < 0.010), although the differences in correlations of negative interpersonal dependent events and negative non-interpersonal events with reassurance-seeking behaviors were not significant (*q* = 0.10, *z* = 1.65, *p* = .099). Moreover, the correlations of negative non-interpersonal dependent events with inattention were stronger than those of the other two types of negative events (*qs* > 0.18, *zs* > 3.01, *ps* < 0.003), and the correlation of negative interpersonal dependent events with inattention was stronger than that of negative independent events (*q* = 0.23, *z* = 3.40, *p* < .001). Finally, the differences in correlations with the lack of perseverance among the three subscales were not significant (*qs* < 0.14, *zs* < 2.14, *ps* > 0.033). Table S3 showed detailed results of the *Z* tests.

**Table 5:** Negative independent events items (English translations)

	Reassurance-seeking behaviors			Inattention		
The neighborhood was noisy.	0.16		[0.04, 0.28]	0.14		[0.02, 0.26]
I got into trouble with my relatives.	0.18		[0.06, 0.31]	0.17		[0.05, 0.30]
I was hit by a disaster such as heavy rain or heavy snow.	0.15		[0.03, 0.27]	0.07		[–0.05, 0.20]
I had to wait for a long time at government offices, stores, banks, etc.	0.12		[–0.01, 0.24]	0.25		[0.13, 0.37]
I was treated coldly at government offices, stores, banks, etc.	0.12		[–0.00, 0.25]	0.02		[–0.11, 0.15]
My family member or a close person were ill, injured, or died.	0.13		[0.01, 0.26]	0.11		[–0.02, 0.23]
I was the victim of crime including theft and molester.	0.07		[–0.06, 0.19]	0.02		[–0.10, 0.15]
Others did something insane to me.	0.26		[0.14, 0.38]	0.29		[0.18, 0.41]
The event that I was looking forward to was canceled or postponed.	0.16		[0.03, 0.28]	0.20		[0.08, 0.32]
I was forced to practice hard in club or circle activities.	0.12		[–0.01, 0.24]	0.11		[–0.01, 0.24]
University facilities such as cafeterias and toilets were inconvenient.	0.25		[0.13, 0.37]	0.23		[0.12, 0.35]
My friend or a lover have moved far away.	0.06		[–0.06, 0.19]	–0.02		[–0.15, 0.11]
The product I purchased was defective.	0.16		[0.03, 0.28]	0.19		[0.06, 0.32]
Many exams and assignments were imposed in my classes.	0.21		[0.09, 0.33]	0.32		[0.21, 0.43]
The household budget in my family has deteriorated.	0.11		[–0.01, 0.24]	0.19		[0.06, 0.31]
I had to make an unexpected expense.	0.24		[0.12, 0.35]	0.38		[0.27, 0.48]
Classroom discipline such as being quiet during the class was not observed.	0.15		[0.02, 0.27]	0.16		[0.03, 0.28]
I heard that there was a trouble in my family.	0.23		[0.11, 0.35]	0.33		[0.22, 0.44]
Suspensions or delays occurred in public transportation such as trains or buses when I used them.	0.13		[0.01, 0.26]	0.11		[–0.02, 0.23]
There was a traffic jam.	0.09		[–0.04, 0.21]	0.13		[0.01, 0.25]

Note: Items with underlines means those with skewness of 2.00 or more, or kurtosis of 4.00 or more. Items with a gray marker mean that their correlations with reassurance-seeking behaviors and inattention were 0.20 or more. Absolute correlations greater than 0.13 are significant at *p* < .05. Numbers in parentheses indicate 95% confidence intervals

## Discussion

### Validity of Negative Independent/Dependent Events Scale

Before discussing the main findings, we wish to note that Negative Independent/Dependent Events Scale items were not selected using factor analysis. Factor analysis is often used for developing new scales. However, we did not use factor analysis because it is incorrect to assume that a latent construct influences the experiences of negative events (Snyder et al., 2019). Instead, we used experts’ judgments to select the subscale items, a method previously adopted by several studies (e.g., Bouchard & Shih 2013; Hankin et al., 2010; Liu & Kleiman, 2012).

Consistent with Hypothesis 1, all the subscales of negative events showed moderate positive correlations with depressive symptoms. This finding confirmed the assumption that events assessed by all the negative events subscales are stressful. Results also showed that both types of negative dependent events were more strongly correlated with depressive symptoms than negative independent events, although this was not the primary investigation of this study. These results might have been obtained because depressive symptoms generate negative dependent events, but not independent events, as shown in many previous studies (Liu & Alloy, 2010 for review). It was also possible that negative dependent events are more detrimental to depression than negative independent events.

Hasegawa et al. (2022b) conducted a longitudinal study with 8- or 9-week intervals using the Negative Independent/Dependent Events Scale. They demonstrated that baseline depressive symptoms were neither significantly associated with negative interpersonal and non-interpersonal events nor negative independent events experienced during the follow-up period. Hasegawa et al. also showed that although all three negative events categories experienced in the 8 weeks before the baseline were associated with an increase in depressive symptoms at follow-up, the influences of these three negative event categories on depressive symptoms were very similar. These findings cannot explain the present result that negative dependent events were more strongly correlated with depressive symptoms than negative independent events. Therefore, more effort is needed to clarify the mechanisms of person-environment interactions that intensify depressive symptoms.

The present findings are partially consistent with the other hypotheses of this study. Negative interpersonal dependent events had a moderate positive correlation with reassurance-seeking behaviors. However, the magnitude of the correlations with reassurance-seeking behaviors differed significantly only between negative interpersonal dependent events and negative independent events, whereas the magnitude of the correlations between negative interpersonal dependent events and negative non-interpersonal dependent events did not differ significantly. Therefore, Hypothesis 2 was only partially supported.

On the other hand, there was a strong positive correlation of negative non-interpersonal dependent events with inattention, and the correlation of negative non-interpersonal dependent events with inattention was significantly stronger than the correlations of negative interpersonal dependent events and negative independent events with inattention. These results supported Hypothesis 3. However, negative non-interpersonal events showed only a weak correlation with lack of perseverance, which was inconsistent with Hypothesis 4 that predicted a moderate or strong correlation, although the results supported the direction of the hypnotized correlation.

The weak correlation between negative non-interpersonal events and the lack of perseverance might have been caused by the item composition of the lack of perseverance subscale. Eight of ten items in this subscale are reversed items. Analyzing the content of the reversed items suggested that the high variance of their scores might reflect constructs other than the lack of perseverance. For example, one reversed item, “unfinished tasks really bother me” might assess not only the lack of perseverance but also worry about unfinished tasks. Therefore, the reversed items might not be appropriate for assessing the lack of perseverance. In fact, the correlations of each reversed item score for the lack of perseverance and negative non-interpersonal events ranged from –.28 to .24, with a mean score of .06, although correlations of the other two items ranged from .31 to .47with a mean score of .39.

The findings in this study supported Hypotheses 1 and 3 and partially supported Hypothesis 2. In addition, experts specializing in clinical psychology confirmed that all items of the Negative Independent/Dependent Events Scale were appropriate for assessing each category of events. The findings of this study and the judgment of experts indicated the acceptable construct validity of the scale.^5^

However, the magnitude of positive correlation with reassurance-seeking behaviors did not significantly differ between negative interpersonal dependent events and negative non-interpersonal dependent events. This result suggested that the scale’s ability to discriminate between these two event categories might be insufficient, although the magnitude of the correlations with inattention differed significantly between negative interpersonal dependent events and negative non-interpersonal dependent events. As described above, self-report measures have difficulties in collecting detailed contextual information about each negative event, including information on whether these events were interpersonal or not (Liu, 2013). This methodological limitation could lead to reduced discrimination between negative interpersonal dependent events and negative non-interpersonal dependent events.

A self-report measure might discriminate between each subscale if the measure were composed of more appropriate items. Therefore, it is desirable to select highly appropriate items based on their correlations with several validity scales as described below.

### Preliminary items of the short scale

This study selected preliminary items for the short scale based on the distribution of each item score and the correlations with reassurance-seeking behaviors and inattention. This procedure reduced the scale’s items from 59 to 36.

The correlations among all subscales in the short scale were smaller than among all the subscales of the longer scale (see Tables 2 and 6). In addition, as shown in Tables S2 and S3, the differences between the correlation of negative interpersonal dependent events with reassurance-seeking behaviors and the correlations of negative non-interpersonal events and negative independent events with reassurance-seeking behaviors were slightly larger in the short scale than in the long one. The difference between the correlation of negative non-interpersonal dependent events with inattention and the correlation of negative independent events with inattention was also more extensive in the short scale than in the long scale, although the differences between the correlation of negative non-interpersonal dependent events with inattention and the correlation of negative interpersonal dependent events with inattention were slightly smaller in the short scale than in the longer one. These results suggest that the discrimination between the subscales was clearer in the short scale than in the long scale, and the short scale has higher validity than the longer one, although these findings might be methodological artifacts.

**Table 6:** Short Negative Independent/Dependent Events Scale’s correlations with other scales (N = 247)

	1		2		3	
1. Negative interpersonal dependent events (15 items)	–					
2. Negative non-interpersonal dependent events (12 items)	0.66		–			
	[0.59, 0.73]					
3. Negative independent events (9 items)	0.53		0.52		–	
	[0.44, 0.62]		[0.43, 0.61]			
4. Depressive symptoms	0.51		0.45		0.27	
	[0.42, 0.61]		[0.35, 0.55]		[0.15, 0.38]	
5. Reassurance-seeking behaviors	0.49		0.41		0.26	
	[0.39, 0.58]		[0.31, 0.51]		[0.14, 0.38]	
6. Inattention	0.48		0.61		0.29	
	[0.39, 0.58]		[0.53, 0.69]		[0.18, 0.41]	
7. Lack of perseverance	0.16		0.20		0.07	
	[0.04, 0.28]		[0.08, 0.32]		[–0.06, 0.19]	

Note: Absolute correlations that was 0.16 or greater were significant at *p* < .05. Numbers in parentheses indicate 95% confidence intervals

### Limitations

This study was disadvantaged by using only two scales to examine appropriate items in each category of negative events. Future efforts using more scales are needed to examine appropriate negative events items. In addition, this study was conducted under the unusual COVID-19 situation in Japan. During the period of this study, university students experienced restrictions in conducting different behaviors, especially interpersonal relationships. Therefore, the correlations among variables in this study might have been different without the pandemic. Another study should be conducted after the pandemic to reexamine the present findings.

Finally, the scale developed in this study was designed to assess the experiences of negative events in Japanese university students. After considering differences in university systems and self-construal between countries described above, it is unsure whether we can apply this scale to assess negative events experienced by university students in countries other than Japan. We hope that researchers in other countries will investigate whether the Negative Independent/Dependent Events Scale could be applied to university students in their country.

## Conclusions

This study developed a scale for separately assessing negative interpersonal dependent events, negative non-interpersonal dependent events, and negative independent events in Japanese university students that were composed of items with sufficient content validity. This study demonstrated the acceptable construct validity of the new scale, although the discrimination between negative interpersonal dependent events and negative non-interpersonal events was relatively ambiguous. The validity of the negative events scales used in previous stress generation studies has not been investigated for correlations with other scales assumed to be related or unrelated. In contrast, the Negative Independent/Dependent Events Scale has the strength of acceptable construct validity demonstrated by correlations with validity measures. We also selected preliminary items for the short scale, which might be more appropriate for assessing each category of negative events.

This study can be positioned within cognitive-behavioral therapy literature as a study elaborating methods of assessing “activating events” in the ABC model. The Negative Independent/Dependent Events Scale was designed to examine factors resulting in stress generation. However, we can also use this scale to examine environmental factors leading to maladaptive cognitive processes (i.e., “beliefs”) and emotional and behavioral consequences (i.e., “consequence”). We are currently conducting a research project on the causes and consequences of stress generation in Japanese university students by using this scale. A part of the findings of this project was reported in Hasegawa et al. (2022b). We expect that evidence accumulated through studies using the Negative Independent/Dependent Events Scale would identify person-environment interactions mechanisms that intensify depression and contribute to theories and the clinical practice of cognitive-behavior therapy.

## Electronic supplementary material

Below is the link to the electronic supplementary material.

Supplementary Material 1

## Data Availability

This study’s dataset can be found at the Open Science Framework [https://osf.io/6t4u8/].

## Footnotes

1 In this article, *negative events* mean events that are often aversive and cause negative emotions such as sadness, anxiety, and anger in many people. Although the meaning of the word *negative* is relatively ambiguous in the psychological literature, the term of *negative events* has been used in this sense in many literatures on stress (e.g., Hammen 2005; Liu, 2013; Monroe & Harkness, 2005). Therefore, this article used the term of *negative events*.

2 The results of this subsequent longitudinal study were reported in Hasegawa et al. (2022b). The data of the present study and that of Hasegawa et al. (2022b) did not overlap.

3 Cook’s D values cannot be calculated when missing data were handled with multiple imputations. Therefore, these values were calculated with handling missing data with Full Information Maximum Likelihood method.

4 The correlation coefficients obtained in this study indicated that power of correlations in the main analyses were sufficient (see Table 2).

5 Subsequent study using the scale developed in this study showed that aggressive behaviors were significantly associated with increase in negative interpersonal dependent events experienced during 8 weeks follow-up period, but not negative non-interpersonal dependent events and negative independent events (Hasegawa et al., 2022b). These findings supplement the results of this study that showed the acceptable construct validity of the Negative Independent/Dependent Events Scale.

## References

[CR1] Adler LA, Spencer T, Faraone SV, Kessler RC, Howes MJ, Biederman J, Secnik K (2006). Validity of pilot Adult ADHD Self-Report Scale (ASRS) to rate adult ADHD symptoms. Annals of Clinical Psychiatry.

[CR2] Auerbach RP, Eberhart NK, Abela JRZ (2010). Cognitive vulnerability to depression in Canadian and Chinese adolescents. Journal of Abnormal Child Psychology.

[CR3] Beck, A. T., Steer, R. A., & Brown, G. K. (1996). *Manual for the Beck Depression Inventory-II*. Psychological Corporation

[CR4] Belmans E, Bastin M, Raes F, Bijttebier P (2019). Temporal associations between social anxiety and depressive symptoms and the role of interpersonal stress in adolescents. Depression and Anxiety.

[CR5] Bouchard LC, Shih JH (2013). Gender differences in stress generation: Examination of interpersonal predictors. Journal of Social and Clinical Psychology.

[CR6] Bos EH, Bouhuys AL, Geerts E, van Os TWDP, Ormel J (2007). Stressful life events as a link between problems in nonverbal communication and recurrence of depression. Journal of Affective Disorders.

[CR7] Cohen, J. (1988). *Statistical power analysis for the behavioral sciences* (2nd ed.). Lawrence Earlbaum Associates

[CR8] Cyders MA (2013). Impulsivity and the sexes: Measurement and structural invariance of the UPPS-P Impulsive Behavior Scale. Assessment.

[CR9] Cyders MA, Smith GT, Spillane NS, Fischer S, Annus AM, Peterson C (2007). Integration of impulsivity and positive mood to predict risky behavior: Development and validation of a measure of positive urgency. Psychological Assessment.

[CR10] Flynn M, Kecmanovic J, Alloy LB (2010). An examination of integrated cognitive-interpersonal vulnerability to depression: The role of rumination, perceived social support, and interpersonal stress generation. Cognitive Therapy and Research.

[CR11] Flynn M, Rudolph KD (2011). Stress generation and adolescent depression: Contribution of interpersonal stress responses. Journal of Abnormal Child Psychology.

[CR12] Hamilton JL, Alloy LB (2017). Physiological markers of interpersonal stress generation in depression. Clinical Psychological Science.

[CR13] Hammen C (1991). Generation of stress in the course of unipolar depression. Journal of Abnormal Psychology.

[CR14] Hammen C (2005). Stress and depression. Annual Review of Clinical Psychology.

[CR15] Hammen, C., & Shih, J. H. (2008). Stress generation and depression. In K. S. Dobson, & D. J. A. Dozois (Eds.), *Risk Factor in Depression* (pp. 409–428). Academic Press

[CR16] Hankin BL, Abramson LY (2002). Measuring cognitive vulnerability to depression in adolescence: Reliability, validity and gender differences. Journal of Clinical Child and Adolescent Psychology.

[CR17] Hankin BL, Stone L, Wright PA (2010). Corumination, interpersonal stress generation, and internalizing symptoms: Accumulating effects and transactional influences in a multiwave study of adolescents. Development and Psychopathology.

[CR18] Harkness KL, Bagby RM, Stewart JG, Larocque CL, Mazurka R, Strauss JS, Kennedy JL (2015). Childhood emotional and sexual maltreatment moderate the relation of the serotonin transporter gene to stress generation. Journal of Abnormal Psychology.

[CR19] Hasegawa, A., Kunisato, Y., Morimoto, H., Nishimura, H., & Matsuda, Y. (2018). Depressive rumination and urgency have mutually enhancing relationships but both predict unique variance in future depression: A longitudinal study. *Cogent Psychology*, *5*(1), Article 1450919. 10.1080/23311908.2018.1450919

[CR20] Hasegawa A, Matsumoto N, Yamashita Y, Tanaka K, Kawaguchi J, Yamamoto T (2022). Response inhibition deficits are positively associated with trait rumination, but attentional inhibition deficits are not: Aggressive behaviors and interpersonal stressors as mediators. Psychological Research Psychologische Forschung.

[CR21] Hasegawa, A., Oura, S., Yamamoto, T., Kunisato, Y., Matsuda, Y., & Adachi, M. (2022b). Causes and consequences of stress generation: Longitudinal associations of negative events, aggressive behaviors, rumination, and depressive symptoms. *Current Psychology*. Advance online publication. 10.1007/s12144-022-02859-9 10.1007/s12144-022-02859-9 PMC8864461 35221638

[CR22] Hashimoto T (1997). Daigakusei ni okeru taijin stress event bunrui no kokoromi [Categorization of interpersonal stress events among undergraduates]. Japanese Journal of Social Psychology.

[CR23] Hashimoto T (2005). Taijin stressor syakudo no kaihatsu [Development of the new scale of interpersonal stressor]. Studies in Humanities (Shizuoka University).

[CR24] Hisata M, Niwa I (1987). Daigakusei no seikatsu sutoressa sokutei ni kansuru kenkyu: daigakuseiyo seikatsu taiken shakudo no sakusei [A study on measuring life stressors of college students: Development of the college life experiences scale]. Studies in sociology psychology and education.

[CR25] Hittner JB, May K, Silver NC (2003). A Monte Carlo evaluation of tests for comparing dependent correlations. Journal of General Psychology.

[CR26] Joiner TE, Wingate LR, Gencoz T, Gencoz F (2005). Stress generation in depression: Three studies on its resilience, possible mechanism, and symptom specificity. Journal of Social and Clinical Psychology.

[CR27] Kanner AD, Coyne JC, Schaefer C, Lazarus RS (1981). Comparison of two modes of stress measurement: Daily hassles and uplifts versus major life events. Journal of Behavioral Medicine.

[CR28] Katsuya N (2004). Kaiteiban juuyou tasya ni taisuru saikakunin keikou syakudo no shinraisei, datousei no kentou [Reliability and validity of Japanese revised version of reassurance-seeking scale]. The Japanese Journal of Psychology.

[CR29] Kessler RC, Adler L, Ames M, Demler O, Faraone S, Hiripi E, Walters EE (2005). The World Health Organization adult ADHD self-report scale (ASRS): A short screening scale for use in the general population. Psychological Medicine.

[CR30] Kessler RC, Adler LA, Gruber MJ, Sarawate CA, Spencer T, Van Brunt DL (2007). Validity of the World Health Organization Adult ADHD self-report scale (ASRS) screener in a representative sample of health plan members. International Journal of Methods in Psychiatric Research.

[CR31] Kikushima K (1999). Stressor to social support ga tyuugaku-ji no hutoukou keikou ni oyobosu eikyou [The effects of stressor and social support on non-attendance tendency in junior high school students]. The Japanese Journal of Personality.

[CR32] Kikushima K (2002). Daigakusei-you stressor syakudo no sakusei: Stress hannou, social support tono kannkei kara [Development of a scale of stressors in university students: Its relationships with stress responses and social support]. Bulletin of Aichi University of Education (Educational Sciences).

[CR33] Kitayama S, Park H, Sevincer AT, Karasawa M, Uskul AK (2009). A cultural task analysis of implicit independence: Comparing North America, Western Europe, and East Asia. Journal of Personality and Social Psychology.

[CR34] Kohn PM, Lafreniere K, Gurevich M (1990). The Inventory of College Student’s Recent Life Experiences: A decontaminated hassles scale for a special population. Journal of Behavioral Medicine.

[CR35] Kojima, M., & Furukawa, T. (2003). *Manual for the Beck Depression Inventory-II (Japanese translation)*. Nihon Bunka Kagakusha Co., Ltd.

[CR36] Liu RT (2013). Stress generation: Future directions and clinical implications. Clinical Psychology Review.

[CR37] Liu RT, Alloy LB (2010). Stress generation in depression: A systematic review of the empirical literature and recommendations for future study. Clinical Psychology Review.

[CR38] Liu RT, Kleiman EM (2012). Impulsivity and the generation of negative life events: The role of negative urgency. Personality and Individual Differences.

[CR39] Lynam, D. R., Smith, G. T., Whiteside, S. P., & Cyders, M. A. (2006). *The UPPS-P: Assessing five personality pathways to impulsive behavior*. Purdue University. [Unpublished report]

[CR40] Markus HR, Kitayama S (1991). Culture and the self: Implications for cognition, emotion, and motivation. Psychological Review.

[CR41] Miura M, Kawaoka F (2008). Koukousei-you gakkou stressor syakudo (SSS) no sakusei [Development of a school stressor scale for high school students (SSS)]. Japanese Journal of Counseling Science.

[CR42] Monroe SM, Harkness KL (2005). Life stress, the “kindling” hypothesis, and the recurrence of depression: Considerations from a life stress perspective. Psychological Review.

[CR43] Muthén, L. K., & Muthén, B. O. (1998–2017). *Mplus User’s Guide. Eighth Edition*.Muthén & Muthén

[CR44] Nishino Y, Kobayashi S, Kitagawa T (2009). Nichijou stressor ga yokuutsu keikou ni oyobosu eikyou to jiko-kati no yakuwari ni tsuite no juudan-kenkyuu [The effect of daily stressors on the tendency toward depression and the role of self-worth in the psychological stress process: A longitudinal study]. The Japanese Journal of Personality.

[CR45] Norwalk K, Norvilitis JM, MacLean MG (2009). ADHD symptomatology and its relationship to factors associated with college adjustment. Journal of Attention Disorders.

[CR46] Okayasu T, Shimada H, Niwa Y, Mori T, Yatomi N (1992). Tyuugakusei no gakkou stressor no hyouka to stress hannou tono kannkei [The relationship between evaluation of school stressors and stress responses in junior high school students]. The Japanese Journal of Psychology.

[CR47] Park, J., Uchida, Y., & Kitayama, S. (2016). Cultural variation in implicit independence: An extension of Kitayama et al. (2009). *International Journal of Psychology*, *51*(4), 269–278. 10.1002/ijop.12157 10.1002/ijop.12157 25711426

[CR48] Pope DJ (2010). The impact of inattention, hyperactivity and impulsivity on academic achievement in UK university students. Journal of Further and Higher Education.

[CR49] Sakamoto S, Kambara M (1998). A longitudinal study of the relationship between attributional style, life events, and depression in Japanese undergraduates. The Journal of Social Psychology.

[CR50] Schwanz KA, Palm LJ, Brallier SA (2007). Attention problems and hyperactivity as predictors of college grade point average. Journal of Attention Disorders.

[CR51] Shimono Y, Hasegawa A (2018). *Daigakusei no gakugyou ni okeru stress taisyo ga hikikomori-shinwasei ni oyobosu eikyou: Coping houryaku to enjo-yousei koudou wo toriagete* [The impact for coping strategies and help-seeking behaviors on the affinity for “hikikomori” among Japanese university students]. Japanese Journal of Cognitive Therapy.

[CR52] Snyder HR, Friedman NP, Hankin BL (2019). Transdiagnostic mechanisms of psychopathology in youth: Executive functions, dependent stress, and rumination. Cognitive Therapy and Research.

[CR53] Snyder HR, Hankin BL (2016). Spiraling out of control: Stress generation and subsequent rumination mediate the link between poorer cognitive control and internalizing psychopathology. Clinical Psychological Science.

[CR54] Starr LR, Hammen C, Brennan PA, Najman JM (2012). Serotonin transporter gene as a predictor of stress generation in depression. Journal of Abnormal Psychology.

[CR55] Stroud CB, Sosoo EE, Wilson S (2018). Rumination, excessive reassurance seeking, and stress generation among early adolescent girls. The Journal of Early Adolescence.

[CR56] Takahashi S (2013). Taijin stressor syakudo sakusei no kokoromi [Development of the Interpersonal Stressor Scale]. The Japanese Journal of Personality.

[CR57] Takahira M (1998). Taijin Tassei ryoikibetsu life event syakudo (daigakusei-you) no sakusei to datousei no kentou [Construction of a scale of life events in interpersonal and achievement domains for undergraduate students]. Japanese Journal of Social Psychology.

[CR58] Takeda T, Tsuji Y, Kurita H (2017). Psychometric properties of the Japanese version of the Adult Attention-Deficit Hyperactivity Disorder (ADHD) Self-Report Scale (ASRS-J) and its short scale in accordance with DSM-5 diagnostic criteria. Research in Developmental Disabilities.

[CR59] Toyama M, Sakurai S (1999). Daigakusei ni okeru nichijou-teki dekigoto to kenkou joutai no kannkei: Positive na nichijou-teki dekigoto no eikyou wo tyuusin ni [Daily hassles and uplifts, and college students’ health: Focusing on daily uplifts]. Japanese Journal of Educational Psychology.

[CR60] West, S. G., Finch, J. F., & Curran, P. J. (1995). Structural equation models with nonnormal variables: Problems and remedies. In R. H. Hoyle (Ed.), *Structural equation modeling: Concepts, issues, and applications* (pp. 56–75). Sage Publications, Inc

[CR61] Whiteside SP, Lynam DR, Miller JD, Reynolds SK (2005). Validation of the UPPS impulsive behaviour scale: A four-factor model of impulsivity. European Journal of Personality.

