# Supplementary material for: Preliminary validation of the self-report measure assessing experiences of negative independent and dependent event frequency in Japanese university students
Source: J Ration Emot Cogn Behav Ther. 2022 Jul 30:1–23. Online ahead of print. doi: 10.1007/s10942-022-00469-9 (PMC9362106; doi:10.1007/s10942-022-00469-9)
Supplement: Supplementary file 1 — Supplementary Material 1 [file 10942_2022_469_MOESM1_ESM.docx]

**Table S1**

*Descriptive statistics of gender and gender differences in scale scores.*

|  | Male | | | | | |  | Female | | | | | |  |  |
| --- | --- | --- | --- | --- | --- | --- | --- | --- | --- | --- | --- | --- | --- | --- | --- |
|  | *n* | | *M* | | *SD* | |  | *n* | | *M* | | *SD* | | *t* | |
| Negative interpersonal dependent events | 147 |  | 37.88 |  | 11.21 |  |  | 92 |  | 40.61 |  | 10.99 |  | 1.85 |  |
| Negative non-interpersonal dependent events | 151 |  | 24.54 |  | 6.29 |  |  | 91 |  | 26.33 |  | 5.52 |  | 2.24 | * |
| Negative independent events | 149 |  | 31.83 |  | 7.83 |  |  | 93 |  | 33.70 |  | 7.20 |  | 1.87 |  |
| Depressive symptoms | 149 |  | 12.29 |  | 9.87 |  |  | 90 |  | 15.93 |  | 11.54 |  | 2.59 | * |
| Reassurance-seeking behaviors | 150 |  | 21.43 |  | 7.63 |  |  | 93 |  | 24.44 |  | 8.53 |  | 2.86 | ** |
| Inattention | 148 |  | 14.84 |  | 5.57 |  |  | 93 |  | 17.08 |  | 5.85 |  | 2.97 | ** |
| Lack of perseverance | 152 |  | 23.47 |  | 5.13 |  |  | 93 |  | 24.13 |  | 4.51 |  | 1.02 |  |
| * *p* < .05, ** *p* < .01. | | | | | | | | | | | | | | | |

**Table S2**

*Differences in correlations with validity measures among Negative Independent/Dependent Events Scale subscales.*

|  | Negative interpersonal dependent events vs. Negative non-interpersonal dependent events | | | |  | Negative interpersonal dependent events vs. Negative independent events | | | |  | Negative non-interpersonal dependent events vs. Negative independent events | | | |
| --- | --- | --- | --- | --- | --- | --- | --- | --- | --- | --- | --- | --- | --- | --- |
|  | *q* | | *Z* | |  | *q* | | *Z* | |  | *q* | | *Z* | |
| Depressive symptoms | 0.02 |  | 0.34 |  |  | 0.15 |  | 2.73 | ** |  | 0.17 |  | 3.01 | ** |
| Reassurance-seeking behaviors | 0.04 |  | 0.76 |  |  | 0.15 |  | 2.67 | ** |  | 0.10 |  | 1.87 |  |
| Inattention | 0.22 |  | 3.84 | *** |  | 0.09 |  | 1.59 |  |  | 0.31 |  | 5.32 | *** |
| Lack of perseverance | 0.06 |  | 1.18 |  |  | 0.06 |  | 1.20 |  |  | 0.12 |  | 2.32 | * |
| * *p* < .05, ** *p* < .01, *** *p* < .001. | | | | | | | | | | | | | | |

**Table S3**

*Differences in correlations* *with validity measures among short Negative Independent/Dependent Events Scale subscales.*

|  | Negative interpersonal dependent events vs. Negative non-interpersonal dependent events | | | |  | Negative interpersonal dependent events vs. Negative independent events | | | |  | Negative non-interpersonal dependent events vs. Negative independent events | | | |
| --- | --- | --- | --- | --- | --- | --- | --- | --- | --- | --- | --- | --- | --- | --- |
|  | *q* | | *Z* | |  | *q* | | *Z* | |  | *q* | | *Z* | |
| Depressive symptoms | 0.08 |  | 1.44 |  |  | 0.29 |  | 4.40 | *** |  | 0.21 |  | 3.14 | ** |
| Reassurance-seeking behaviors | 0.10 |  | 1.65 |  |  | 0.27 |  | 4.01 | *** |  | 0.17 |  | 2.58 | ** |
| Inattention | 0.18 |  | 3.02 | ** |  | 0.23 |  | 3.40 | *** |  | 0.41 |  | 5.96 | *** |
| Lack of perseverance | 0.04 |  | 0.77 |  |  | 0.09 |  | 1.49 |  |  | 0.14 |  | 2.13 | * |
| * *p* < .05, ** *p* < .01, *** *p* < .001. | | | | | | | | | | | | | | |
